# The Effect of Heat Treatment on the Microstructure and Mechanical Properties of the Novel Low-Cost Ti-3Al-5Mo-4Cr-2Zr-1Fe Alloy

**DOI:** 10.3390/ma13173798

**Published:** 2020-08-28

**Authors:** Meng Sun, Dong Li, Yanhua Guo, Ying Wang, Yuecheng Dong, Zhenhua Dan, Hui Chang

**Affiliations:** 1 Tech Institute for Advanced Materials & College of Materials Science and Engineering, Nanjing Tech University, Nanjing 210009, China; sun2018@njtech.edu.cn (M.S.); binhaiman@163.com (D.L.); dongyuecheng@njtech.edu.cn (Y.D.); zhenhuadan@njtech.edu.cn (Z.D.); ch2006@njtech.edu.cn (H.C.); 2 Jiangsu Collaborative Innovation Center for Advanced Inorganic Function Composites, Nanjing Tech University, Nanjing 210009, China; 3 Chengdu Advanced Metal Materials Industrial Technology Research Institute Co. Ltd., Chengdu 610000, China; wangying2205b@163.com

**Keywords:** titanium alloy, heat treatment, microstructure, mechanical properties

## Abstract

In order to reduce the cost of titanium alloys, a novel low-cost Ti-3Al-5Mo-4Cr-2Zr-1Fe (Ti-35421) titanium alloy was developed. The influence of heat treatment on the microstructure characteristics and mechanical properties of the new alloy was investigated. The results showed that the microstructure of Ti-35421 alloy consists of a lamina primary α phase and a β phase after the solution treatment at the α + β region. After aging treatment, the secondary α phase precipitates in the β matrix. The precipitation of the secondary α phase is closely related to heat treatment parameters—the volume fraction and size of the secondary α phase increase when increasing the solution temperature or aging time. At the same solution temperature and aging time, the secondary α phase became coarser, and the fraction decreased with increasing aging temperature. When Ti-35421 alloy was solution-treated at the α + β region for 1 h with aging surpassing 8 h, the tensile strength, yield strength, elongation and reduction of the area were achieved in a range of 1172.7–1459.0 MPa, 1135.1–1355.5 MPa, 5.2–11.8%, and 7.5–32.5%, respectively. The novel low-cost Ti-35421 alloy maintains mechanical properties and reduces the cost of materials compared with Ti-3Al-5Mo-5V-4Cr-2Zr (Ti-B19) alloy.

## 1. Introduction

Titanium alloys are widely used in aerospace, biomedical, and marine engineering due to their low density, excellent mechanical properties and good corrosion resistance [1,2,3,4]. In series of titanium alloys, near-β titanium alloys have a good combination of strength–ductility–toughness, and can obtain a better hot processing performance than most industrial titanium alloys [5]. However, the production cost of near-β alloys is too high due to the use of expensive alloying elements (V, Zr, Nb, Mo, etc.), which limits the wider application of these alloys. Hence, many efforts were made to reduce the cost of materials by replacing these expensive alloying elements with cheaper alloying elements, such as Fe, Cr, Mn, etc. [6,7,8] Ti-B19 alloy [9,10] is a novel near-β titanium alloy developed by Northwest Institute for Non-ferrous Metal Research, China. After heat treatment, the ultimate strength, elongation and toughness of Ti-B19 alloy are 1250 MPa, 7.7% and 70 MPa·m^1/2^, respectively. The expensive β-stabilizers (Mo, V and Cr) contained in Ti-B19 alloy provide it with high toughness and strength [11,12]; they also make the material cost of the alloy very high. Fe is very cheap and it has been reported that Fe-alloyed titanium can obtain a better balance between strength and ductility, such as in the cases of Ti-5Al-5Mo-5V-3Cr-0.5Fe [13] and Ti-5Al-3Mo-3V-2Cr -2Zr-1Nb-1Fe [14], while when 3% iron is added, the iron in the titanium alloy is easy to segregate [15]. To reduce the cost and maintain the mechanical properties of Ti-B19 alloy, an inexpensive Fe was added to replace the expensive V. According to the equation of Mo equivalence [14] ([Mo]_eq_): [Mo]_eq_ = [Mo] + 0.2[Ta] + 0.28[Nb] + 0.4[W] + 0.67[V] + 1.25[Cr] + 1.25[Ni] + 1.7[Mn] + 1.7[Co] + 2.5[Fe], the [Mo]_eq_ of Ti-B19 is 12.68. In order to make the Mo equivalence of the new alloy the same as Ti-B19 alloy, 1 wt% Fe was added to replace 4 wt% V to maintain the [Mo]_eq_. The nominal composition of the new designed alloy is Ti-3Al-5Mo-4Cr-2Zr-1Fe.

The microstructure of near-β titanium alloys can be significantly improved through heat treatment, and the microstructures have a decisive influence on the properties of these titanium alloys [16,17,18,19]. For example, Ti-55531 [20] alloy was solution-treated at 880 °C for 1.5 h and aged at 620 °C for 10 h; the equiaxed primary α phases distribute at the β grain boundaries, and secondary α phases irregularly precipitate inside the β grains. The tensile strength, yield strength and elongation of Ti-55531 are 1178 MPa, 1100 MPa and 9%, respectively. It has been reported that Ti-7333 alloy [21] and Ti-5321 alloy [14] undergo reasonable heat treatment; both alloys can obtain excellent microstructure and mechanical properties. Hence, the influence of heat treatment on the microstructure characteristics and mechanical properties of Ti-35421 alloys needs to be investigated. In this study, different heat treatments were chosen to examine the evolution of the microstructure and tensile properties of Ti-35421 alloy.

## 2. Experiment

**Figure 1:**
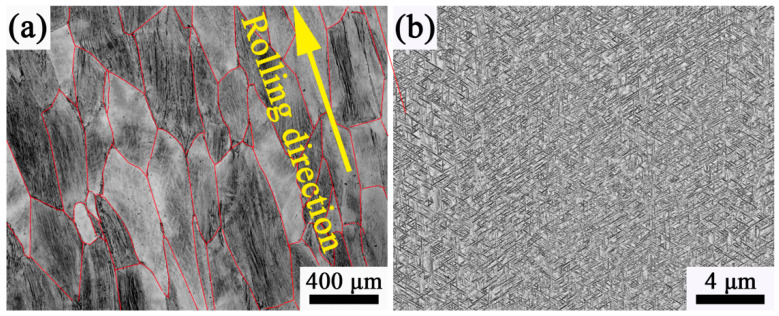
Microstructure of the as-rolled specimen: (**a**) Optical micrograph; (**b**) Scanning Electron micrograph.

**Figure 2:**
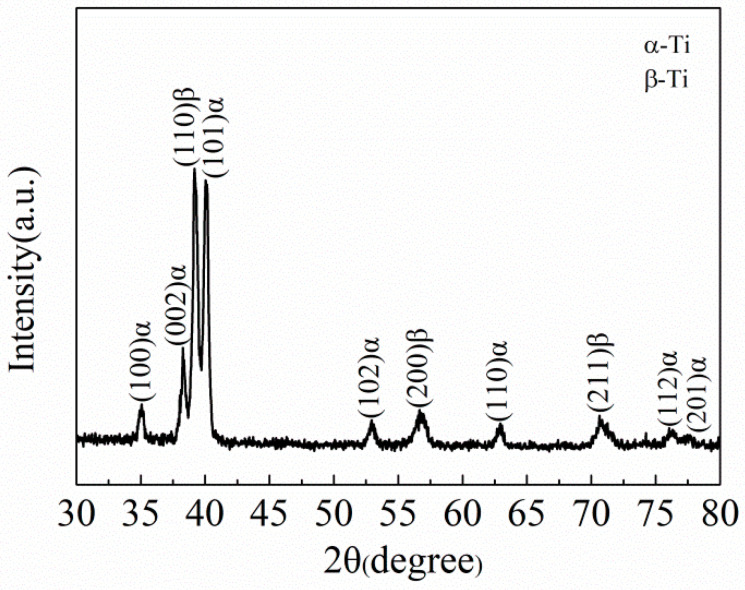
X-ray diffraction spectra of the as-rolled specimen.

The Ti-35421 alloy ingot was fabricated by a thrice vacuum consumption arc melting process using high-purity sponge titanium, Al-80Mo alloy, Al-85V alloy, high-purity aluminum wire, high-purity chromium, high-purity iron, and high-purity zirconium. The composition of the alloy ingot (wt%) is aluminum: 3.04; molybdenum: 5.01; chromium: 3.80; zirconium: 1.88; iron: 1.00; oxygen: 0.09; and the balance is titanium. After melting, the ingot was forged and rolled at 1050 °C and 900 °C, respectively, then cut into 22 × 83 × 510 mm^3^ alloy sheets. The microstructure of the as-rolled sample is shown in Figure 1. The grains are coarse crystal, and along the rolling direction, the average grain size is about 320 μm, and the aspect ratio is about 2:1. The microstructure of the alloy consists of the β and α phase, and the β grain boundary shape is irregular. Figure 2 shows the XRD patterns of the as-rolled alloy. As can be seen, the structure of the as-rolled alloy consists of the β phase and the α phase.

Near-β titanium alloys are generally subjected to a short-time solution treatment in the α + β region or the β region and the aging range between 500 °C and 600 °C for different times to satisfy the engineering requirements [22]. The β-transformation temperature of Ti-35421 alloy is about 803 ± 5 °C, as measured by the metallographic method. Therefore, the solution temperature is selected at 760 °C and 780 °C for 1 h and 820 °C and 840 °C for 0.5 h, followed by being air-cooled at room temperature. After solution treatment, the specimens are aged at 500 °C, 520 °C, 540 °C and 560 °C for 8 h, respectively, with air-cooling. The experimental results show that the alloy matched the strength and plasticity when the alloy was solution-treated at 780 °C for 1 h and aged at 540 °C for 8 h. Therefore, the solution temperature was selected at 760 °C and 780 °C for 1 h and 820 °C and 840 °C for 0.5 h, followed by air-cooling to room temperature. After solution treatment, the specimens were aged at 500 °C, 520 °C, 540 °C and 560 °C for 8 h with air-cooling, respectively. The results indicate that the alloy obtained excellent mechanical properties when solution-treated at 780 °C for 1 h and aged at 540 °C for 8 h. Therefore, the solution and the soaking temperature of the aging treatment were set to this parameter. In order to investigate the influence of aging time on the alloy, the aging time ranges from 2 h to 16 h with air-cooling in this study.

The microstructure of the specimen was observed by Optical microscopy (OM, Axio Observer A1m, ZEISS, Oberkochen, Germany) and Scanning Electron Microscope (SEM, FEI, Scios, Waltham, MA, USA). The specimen for OM observation was polished with SiC paper and diamond paste (0.5 μm), then etched with Kroll solution (5 mL HF, 10 mL HNO_3_ and 70 mL H_2_O). The specimen for SEM observation was polished with SiC paper and colloidal silica (OP-S). The phase and fraction of the precipitates were measured using Image-Pro Plus software (ver 6.0, Media Cybernetics, MD, USA). The phase composition of Ti-35421 alloy was analyzed by an X-ray diffractometer (XRD, DMAX-RB, Rigaku, Japan).

After heat treatment, specimens for the tensile tests were machined into a cylindrical shape with a 25.0 mm gauge length and a 5.0 mm in diameter. Room temperature tensile tests of samples were carried out using the universal tensile testing machine (ETM205D, Wance, Shenzhen, China) with an applied strain rate of 1 × 10^−3^ s^−1^.

## 3. Results and Discussion

### 3.1. Microstructure of Ti-35421 Alloy

#### 3.1.1. Microstructure of As-Solutionized States

**Figure 3:**
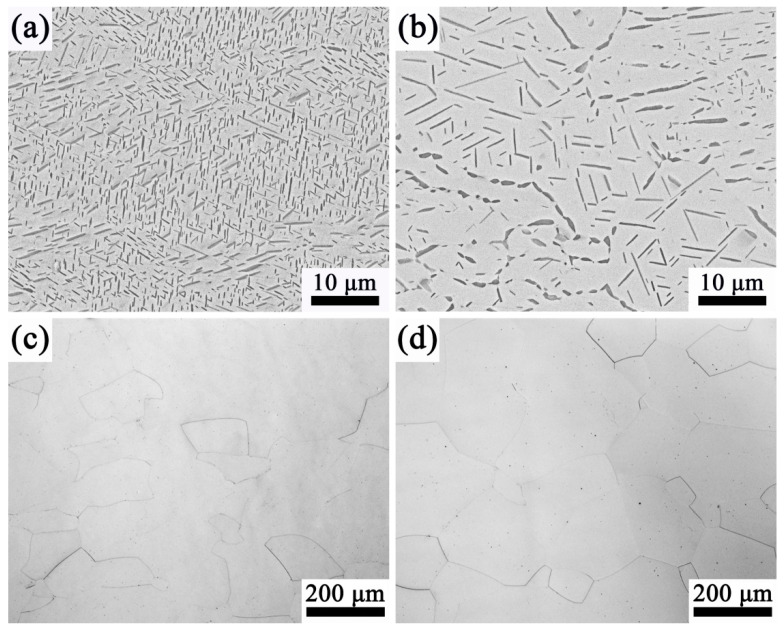
Micrographs of the specimen that was solution-treated at different temperatures: (**a**) 760 °C for 1 h; (**b**) 780 °C for 1 h; (**c**) 820 °C for 0.5 h; (**d**) 840 °C for 0.5 h.

Figure 3 shows the microstructure of the specimen that was solution-treated at different temperatures with air-cooling. As can be seen, after solution treatment at the α + β region (Figure 3a,b), the lamina α phase is distributed on the β matrix uniformly, and there is no orientation relationship between the two phases. When solution-treated at the β region, β grains become coarse and no α phase can be observed (Figure 3c,d). At 840 °C, the microstructure is composed of large β grains with a size of ~200 μm. This phenomenon indicates that the pinning effect of the primary α phase limits the growth tendency of β grains when the alloy is solution-treated at the α + β region [23]. With increasing solution temperature, the primary α phase transforms into the β phase, and the pinning effect of the primary α phase gradually weakens. When the alloy was solution-treated at the β region, the α phase dissolves completely, and the pinning effect disappeared, causing the β phase to grow larger (Figure 3c,d).

**Figure 4:**
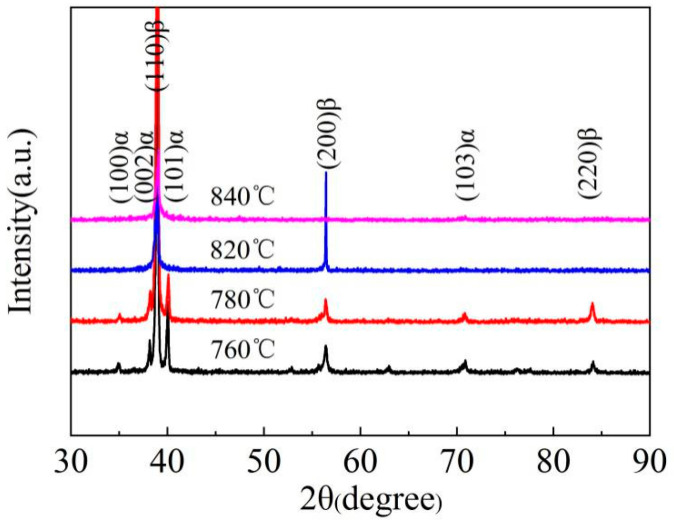
X-ray diffraction spectra of specimens after different solution treatments.

The microstructure of Ti-35421 alloy at different solution temperatures also indicates that the volume fraction of the primary α phase varies with the temperature. Figure 3a is obtained by solution treatment at 760 °C. It can be found that the volume fraction of the α phase decreases by comparing the microstructure after hot rolling (Figure 1b). When the solution temperature increased from 760 °C to 780 °C, the volume fraction of the α phase decreased from 21.4% (Figure 3a) to 14.5% (Figure 3b). When the solution temperature reached 820 °C (Figure 3c), the primary α phase dissolved completely. Figure 4 shows the XRD patterns of alloys with different solution temperatures. The result shows that the microstructure of Ti-35421 alloy consists of an α phase and a β phase after solution treatment. When the solution temperature exceeds the β-transformation temperature, only the peaks of (110) β and (200) β exist. After the solution temperature below the β-transformation temperature, a series of α phase and (200) β peaks exist. The analysis results are consistent with the metallographic analysis results of the alloy.

#### 3.1.2. Microstructure of Aged Alloys

**Figure 5:**
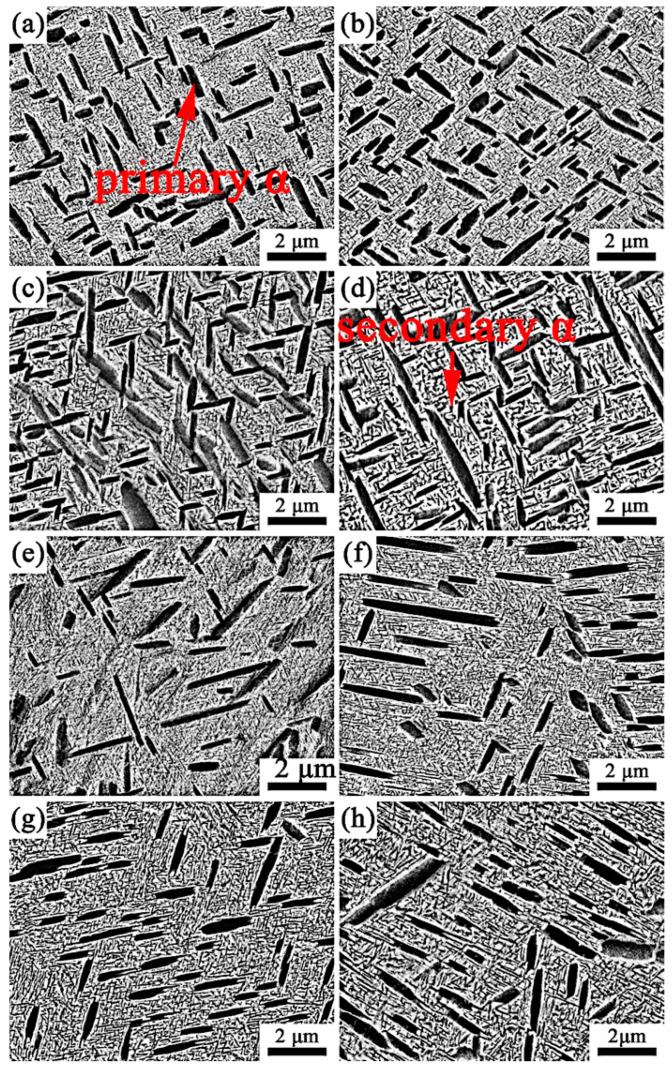
Microstructures of the alloy that was solution-treated for 1 h and aged for 8 h: (**a**) 760 °C + 500 °C; (**b**) 760 °C + 520 °C; (**c**) 760 °C + 540 °C; (**d**) 760 °C + 560 °C; (**e**) 780 °C + 500 °C; (**f**) 780 °C + 520 °C; (**g**) 780 °C + 540 °C; (**h**) 780 °C + 560 °C.

**Figure 6:**
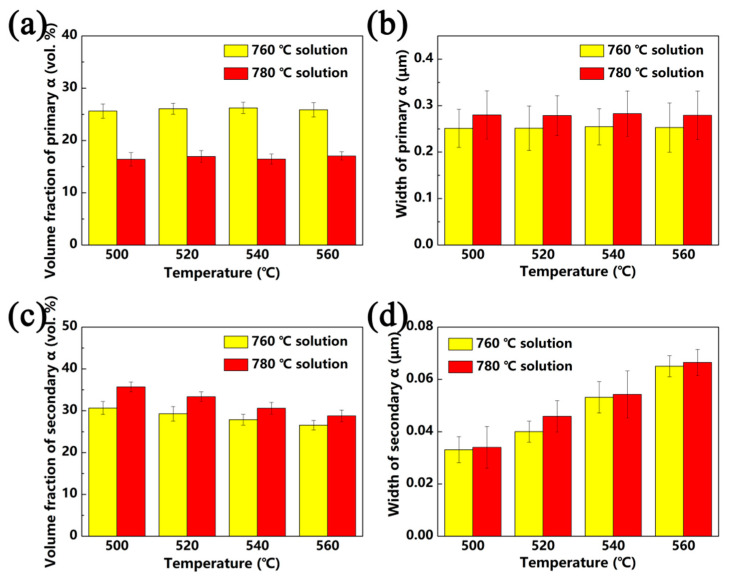
Microstructure features of the α phase that was solution-treated at the α + β region for 1 h and aged for 8 h: (**a**) volume fraction of primary α; (**b**) width of primary α; (**c**) volume fraction of secondary α; (**d**) width of secondary α.

The microstructure evolution of Ti-35421 alloy when solution-treated at α + β regions with different aging treatments is investigated. Figure 5 shows the SEM micrographs of specimens that were solution-treated at 760 °C and 780 °C for 1 h with aging treatments of from 500 °C to 560 °C for 8 h. It can be seen that microstructure of specimens consist of a β matrix, a lamina primary α phase and a fine lamellar secondary α phase, and a secondary α phase, which are distributed homogeneously. When the specimen was solution-treated at the α + β region (Figure 5a–d), with increasing aging temperature, the morphology and volume fraction of the primary α phase in the β matrix changed slightly, but the size and volume fraction of the secondary α phase changed significantly. The volume fraction and width of the secondary α phases and primary α phases are summarized in Figure 6. Only the width of the secondary α phase is measured due to the size of the β grain in the structure limits of the length of the secondary α phase [24].

As shown in Figure 6, the volume fraction of the secondary α phase that was solution-treated at 780 °C and aged at 500 °C is about 35.7%. By increasing the aging temperature to 560 °C, the volume fraction of the secondary α phase decreased to 28.8%, with the width of the secondary α phase increased from 34 nm to 66.5 nm (Figure 6c,d). The same trend was observed with solution treatment at 760 °C. With increasing aging temperature, the secondary α phase grows gradually, while the volume decreases. The β phase may transform into the α phase after solution treatment in combination with aging treatment due to the Gibbs free energy difference of the two phases. The lower the aging temperature, the stronger the driving force of the phase transformation [25,26]. When the alloys was aged at low temperatures (500 °C), the phase transformation driving force of β/α was larger, allowing secondary α phase nucleates to form easily. However, the diffusion of elements in the alloy is difficult at low temperatures, meaning that the secondary α phase is difficult to grow. As a result, the smaller secondary α phase precipitates in the β phase matrix. When the alloys were aged at a higher temperature (560 °C), the phase transformation driving force of β/α was smaller, and the secondary α phase nucleation was difficult. However, the higher aging temperature makes the diffusion of the alloy element easier, and the secondary α phases show a trend of growing larger.

**Figure 7:**
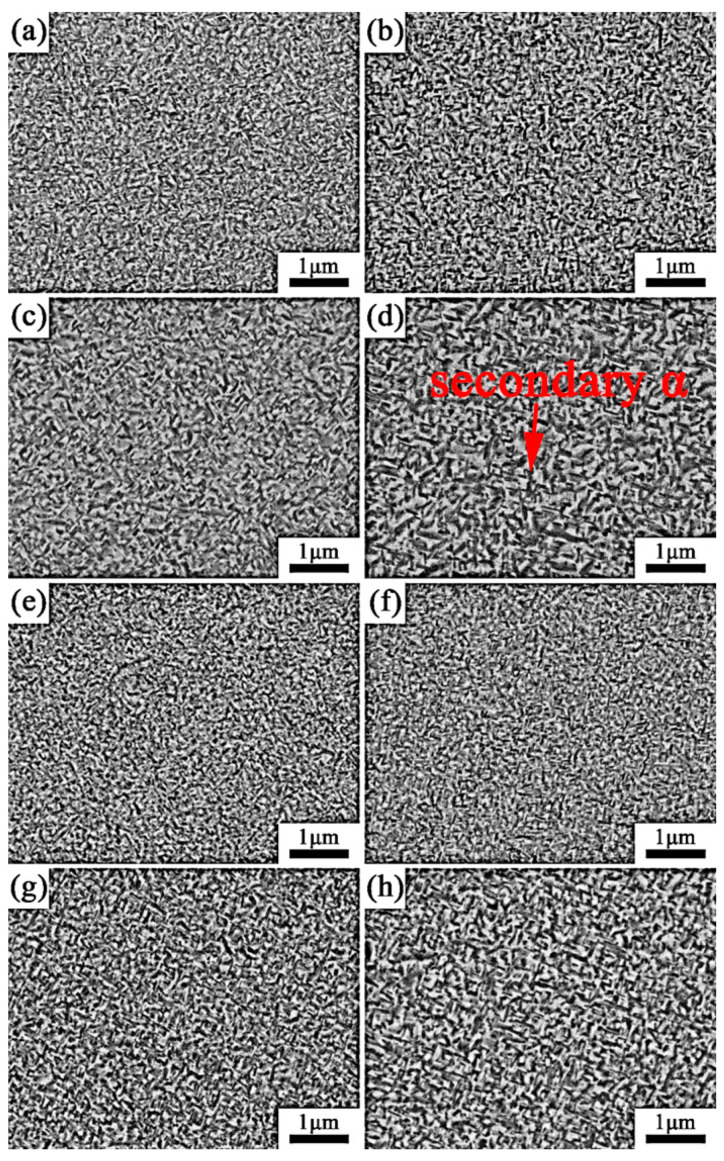
Microstructures of the alloy after solution treatment for 0.5 h and aged for 8 h: (**a**) 820 °C + 500 °C; (**b**) 820 °C + 520 °C; (**c**) 820 °C + 540 °C; (**d**) 820 °C + 560 °C; (**e**) 840 °C + 500 °C; (**f**) 840 °C + 520 °C; (**g**) 840 °C + 540 °C; (**h**) 840 °C + 560 °C.

**Figure 8:**
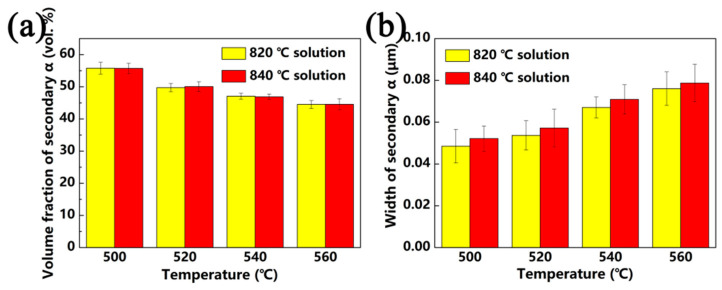
Microstructure features of the α phase that was solution-treated at the β region for 1 h and aged for 8 h: (**a**) volume fraction of secondary α; (**b**) width of secondary α.

Figure 7 shows the SEM micrographs of specimens that were solution-treated at 820 °C and 840 °C for 1 h with aging treatment from 500 °C to 560 °C for 8 h. After being solution-treated at the β region with aging, a significant number of fine lamellar secondary α phases precipitated uniformly within the β matrix. Thus, it is free of the primary α phase. As seen from Figure 8, with a solution treatment of 820 °C for 0.5 h, followed by an aging treatment of 500 °C for 8 h, the volume fraction and width of the secondary α phase are 55.5% and 48.5 nm, respectively. With the aging temperature increasing to 560 °C, the volume fraction and width of the secondary α phase change to 44.2% and 76 nm, respectively, as shown in Figure 8. Under the same solution conditions, secondary α starts to coarsen, and the volume fraction decreases gradually when the aging temperature increases.

The size and volume fractions of secondary α are not only related to the aging temperature, but also the solution temperature. After being solution-treated at 760 °C plus aging at 500 °C, a number of fine lamellar secondary α phases precipitated in the matrix and were distributed uniformly. As the solution temperature increases to 780 °C, the width of the secondary α phase increases, and the volume fraction decreases. When the alloy was solution-treated at the β region with aging treatment, the primary α phase dissolved into the β matrix. Secondary α is easier to nucleate and grow due to the higher driving force for subsequent and lower stable matrix composition [27]. With a continuous increase in solution temperature, only a slight change took place in the secondary α phase.

**Figure 9:**
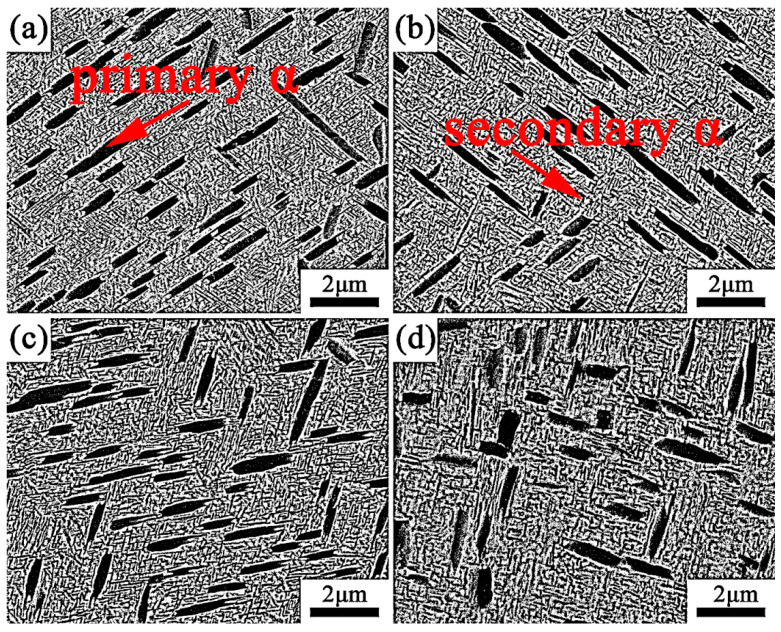
Microstructures of the alloy after solution treatment at 780 °C for 1 h and aging at 540 °C for different lengths of time: (**a**) 2 h; (**b**) 4 h; (**c**) 8 h; (**d**) 16 h.

**Figure 10:**
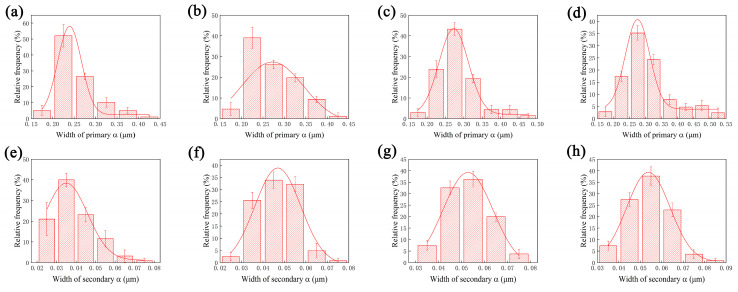
The primary and secondary width distributions of Ti-35421 that was solution-treated at 780 °C for 1 h and aged at 540 °C for different lengths of time time: (**a**,**e**) 2 h; (**b**,**f**) 4 h; (**c**,**g**) 8 h; (**d**,**h**) 16 h.

Figure 9 shows microstructure of the alloy after being solution-treated at 780 °C and aging at 540 °C with different lengths of treatment time. As indicated in Figure 9, lamina primary α and lamellar secondary α are distributed in the β matrix. After aging treatment for different lengths of time, a slight change took place in the volume fraction of the α phase, as shown in Table 1. However, the α phase displayed a growing trend with increasing aged time (Figure 10), and the proportion of the wider α gradually increased with increasing aging time. At 2 h, fine lamellar secondary α precipitated in the matrix with a width of 40 nm. When aging time reached 8 h, secondary α began to clearly coarsen with a width of 54 nm. Furthermore, primary α also began to coarsen (Figure 9c and Figure 10c). With continuously increasing aging time, secondary α showed slight changes in morphology and size. The effect of aging time was weaker than the effect of aging temperature.

### 3.2. Mechanical Properties of the Alloy after Heat Treatment

#### 3.2.1. Mechanical Properties of Solution-Treated Alloys

Generally, near-β titanium alloys require solution and age treatment to achieve ideal properties. The solution’s primary function is to eliminate the heterogeneity of structure and make the solute atoms dissolve back into the β matrix to prepare for the aging treatment of the alloy.

**Figure 11:**
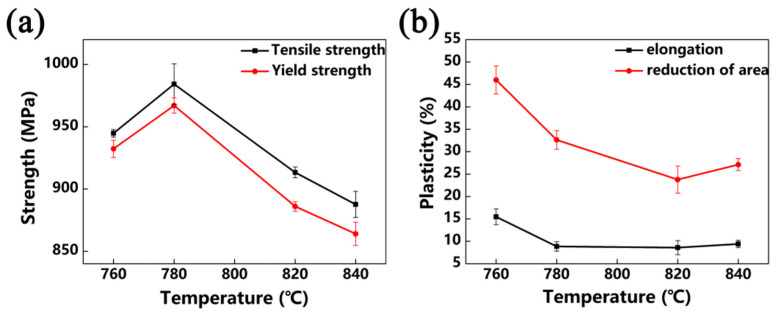
Tensile properties of the alloy that was solution-treated at different temperatures for 8 h: (**a**) tensile strength and yield strength of the alloy; (**b**) elongation and reduction of area of the alloy.

Figure 11 shows the tensile properties of Ti-35421 alloy under different solution treatments. It is clear that the tensile strength of Ti-35421 alloy is about 930 MPa, and elongation is between 8.5% and 15.1%. When the alloy was solution-treated at the α + β region, the alloy shows a higher tensile strength (960 MPa) than that above trans temperature (900 MPa). This phenomenon can be explained by the pinning effect of the primary α phase that limits the growth of β grains [23]. In the case of 760 °C, the alloy’s microstructure consists of the lamina α phase and β matrix (Figure 3a), and the alloy has the greatest elongation (15.1%). When the solution temperature increases to 780 °C, the tensile strength of the alloy reaches its peak (968 MPa). When the solution temperature increases to 780 °C, the solution atomic concentration rises in the β matrix due to the primary α phase dissolving, which results in an increase in the tensile strength and a decrease in elongation. When the alloy was solution-treated at the β region, the primary α phase was completely dissolved in the β matrix, and the pinning effect of the primary α phase disappeared, leading to the recrystallization of β grains. The alloy’s strength decreased, and the ductility improved due to the recrystallization to eliminate residual internal defects and stress relief [28].

#### 3.2.2. Mechanical Properties of Aged Alloys

After solution treatment, Ti-35421 alloy has great elongation, while the strength of the alloy is moderate. It is well known that aging treatment can optimize the balance of strength and elongation.

**Figure 12:**
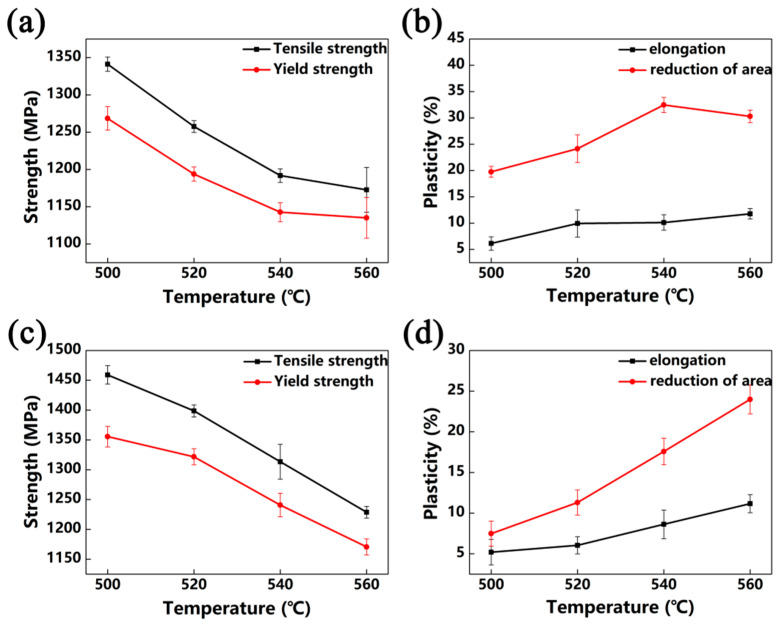
Tensile properties of Ti-35421 that was solution-treated at the α + β region for 1 h with 8 h aging: (**a**,**b**) solution-treated at 760 °C; (**c**,**d**) solution-treated at 780 °C.

**Figure 13:**
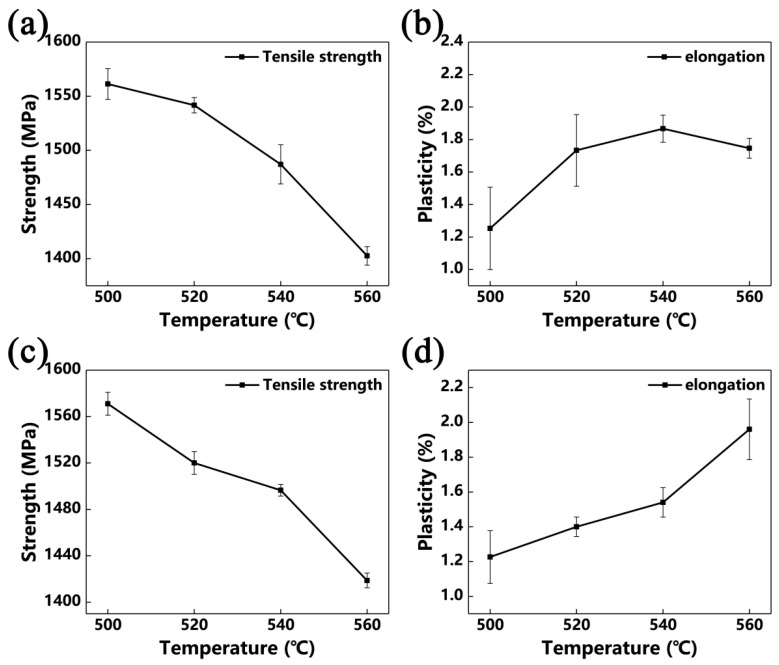
Tensile properties of Ti-35421 that was solution-treated at the β region for 0.5 h and with 8 h aging: (**a**,**b**) solution-treated at 820 °C; (**c**,**d**) solution-treated at 840 °C.

Figure 12 and Figure 13 show the tensile properties of Ti-35421 alloy after different heat treatments. Solution and age temperature affect the properties of Ti-35421 alloy. As can be seen in Figure 12 and Figure 13a,b, the strength of the alloy increases, while ductility decreases with the increase in solution temperature. With a continuous increase in solution temperature of up to 840 °C, only a slight change in properties of the alloy compared to that solution-treated at 820 °C can be observed. The volume fraction, size, morphology, and distribution of the α phase affect the strength of the near-β titanium alloy, and ductility is affected by the volume fraction of the primary α phase and β grain size [29,30]. As shown in Figure 5 and Figure 6, in the case of the alloy that was solution-treated at 760 °C with aging at 500 °C, secondary α was too fine (~33 nm) with a volume fraction of 30.7%, and the primary α phase with a volume fraction of 25.6% was distributed in the matrix. Thus, the ultimate strength, yield strength, elongation, and reduction of area are 1341 MPa, 1269 Mpa, 6.1%, and 19.8%, respectively. Under solution treatment at 780 °C and aging treatment at 500 °C, the volume fraction and width of the secondary α phase are 35.7% and, 34 nm, respectively, and about 9% of primary α is dissolved into the β matrix, causing the strength to increase and ductility to decrease. As compared with the alloy that was solution-treated at the α + β region, more secondary α precipitates, and primary α is dissolved into the β matrix completely when solution-treated at the β region, which leads to the alloy having the highest strength and poorest ductility.

Solution treatment has a significant impact on the properties of near-β titanium alloys, and aging treatment also has a huge effect on the properties. As shown in Figure 12 and Figure 13, it is deduced that the ductility of Ti-35421 alloy increases with the increase in aging temperature, while the strength decreases. This phenomenon can be attributed to the increase in the volume fraction of secondary α allowing for the improvement in the decrease in its size [22,31]. Under the same solution temperature (760 °C), secondary α begins to coarsen as increases the aging temperature (Figure 8), while the volume fraction gradually decreases. Thus, the strength of Ti-35421 alloy decreases from 1341 Mpa to 1173 Mpa, and the ductility increases to 11.78% due to the coarsening of secondary α.

**Figure 14:**
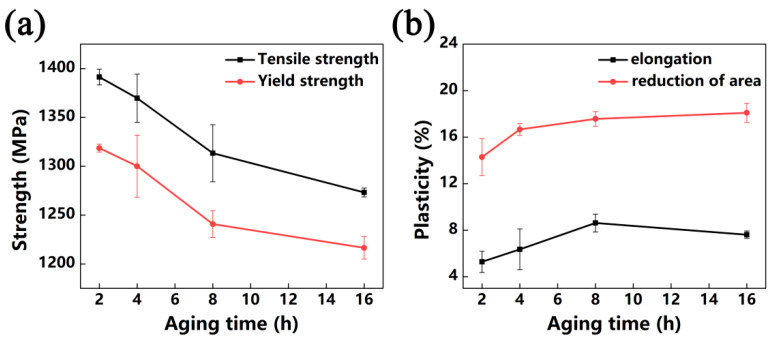
Tensile properties of the alloy was solution-treated at 780 °C and aged at 540 °C for different lengths of time: (**a**) tensile strength and yield strength of the alloy; (**b**) elongation and reduction of area of the alloy.

Aging time is a key parameter in aging treatment. Figure 14 shows that tensile properties of the alloy that was solution-treated at 780 °C and aged at 540 °C with different lengths of time. After aging treatment for 2 h, the width of primary α was 249 nm, and the secondary α was very fine with a width of 40 nm. The alloy has high strength and poor ductility under this condition. By extending aging time, the strength decreases gradually as secondary α grows (Figure 10e–h). In the meantime, the increase in the α volume fraction improves the ductility. When aging time exceeds 8 h, growth and the volume fraction of α begin to slow down; thus, the change in properties is slight.

### 3.3. Comparison with Ti-B19 Alloy

It has been reported that Ti-B19 is a high-strength and tough near-β titanium alloy with good hot workability and weldability [9,10]. As can be seen in Table 2, the tensile strength, yield strength, elongation, and reduction of area of Ti-B19 alloy can reach 1175–1360 MPa, 1120–1325 MPa, 7–10%, and 17–32.5%, respectively. Although the properties of the alloys under different heat treatments are different, it can be found that Ti-35421 alloy has an excellent balance of strength–ductility. After suitable heat treatment, the tensile strength and yield strength of Ti-35421 alloy are superior to Ti-B19 alloy, and the ductility of Ti-35421 alloy can reach the level of Ti-B19. Fe and V are β-stability elements. Lin et al. [32] and Hsu et al. [33] have reported that the grain size of titanium alloy decreases with Fe addition, which can improve the mechanical properties of the alloy. Ti-35421 alloy has low cost and the same mechanical properties as Ti-B19 alloy.

## 4. Conclusions

In this study, the influence of heat treatment on the microstructure characteristics and mechanical properties of Ti-3Al-5Mo-4Cr-2Zr-1Fe alloy has been investigated. The main results are as follows:

The volume fraction of the primary α phase depends on the solution temperature. When Ti-35421 alloy was solution-treated at the α + β region, the microstructure of Ti-35421 alloy consisted of a lamina primary α phase and a β phase. When increasing the solution temperature above the trans temperature, no α phase could be found.

The precipitation of the secondary α phase is closely related to solution temperature, aging temperature and aging time. After aging, the lamellar secondary α phase precipitates in the β matrix. When increasing solution temperature or aging time, both the volume fraction and size of the secondary α phase increase. At the same solution temperature and aging time, the secondary α phase precipitates became coarser, and fraction decreased with increasing aging temperature.

Both solution and aging temperature have a significant effect on the tensile properties of Ti-35421 alloy. With increasing aging temperature, the strength of the alloy decreases, while ductility increases. When the solution-treated at the α + β region for 1 h plus aging surpasses 8 h, Ti-35421 alloy can obtain a better combination of tensile properties when compared with the solution-treated at the β region, and the ultimate strength, yield strength, elongation and reduction of area are in a range of 1172.7–1459.0 MPa, 1135.1–1355.5 MPa, 5.2–11.8% and, 7.5–32.5%, respectively. The influence of aging time on the tensile properties of Ti-35421 alloy is weaker than the aging temperature.

Ti-35421 alloy maintains mechanical properties and reduces the cost of materials when compared with Ti-B19 alloy. The novel near-β titanium alloy brings together low cost and excellent balance of strength and ductility.

## Figures and Tables

**Table 1:** Microstructure features of the α phase that was solution-treated at 780 °C for 1 h plus aging at 540 °C for different lengths of time.

Aging Time (h)	Primary α Phase		Secondary α Phase	
	Average Width (μm)	Volume Fraction (%)	Average Width (μm)	Volume Fraction (%)
2	0.249 ± 0.044	14.87 ± 1.57	0.0401 ± 0.007	28.40 ± 1.01
4	0.257 ± 0.015	15.44 ± 0.99	0.0451 ± 0.009	29.53 ± 1.48
8	0.283 ± 0.069	16.23 ± 1.19	0.0542 ± 0.006	30.61 ± 1.40
16	0.283 ± 0.061	16.43 ± 0.86	0.0567 ± 0.009	31.15 ± 1.73

**Table 2:** Strength–toughness trades of Ti-3Al-5Mo-1Fe -4Cr-2Zr alloy.

Heat Treatment	Tensile Strength (MPa)	Yield Strength (MPa)	Elongation (%)	Reduction of Area (%)
760 °C/1 h	944.8	932.3	15.5	46.0
780 °C/1 h	984.2	967.0	8.8	32.6
820 °C/0.5 h	913.4	886.0	8.6	23.8
840 °C/0.5 h	887.6	864.0	9.4	27.1
760 °C/1 h + 500 °C/8 h	1341.2	1268.5	6.1	19.8
760 °C/1 h + 520 °C/8 h	1268.4	1203.2	9.5	24.3
760 °C/1 h + 540 °C/8 h	1191.7	1142.8	10.1	32.5
760 °C/1 h + 560 °C/8 h	1172.7	1135.1	11.8	30.3
780 °C/1 h + 500 °C/8 h	1459.0	1355.5	5.2	7.5
780 °C/1 h + 520 °C/8 h	1398.7	1321.7	6.0	11.3
780 °C/1 h + 540 °C/2 h	1391.4	1318.6	5.3	14.3
780 °C/1 h + 540 °C/4 h	1369.6	1300.1	6.4	16.7
780 °C/1 h + 540 °C/8 h	1313.4	1240.8	8.6	17.6
780 °C/1 h + 540 °C/16 h	1273.3	1216.5	7.6	18.1
780 °C/1 h + 560 °C/8 h	1228.8	1170.6	11.2	24.0
820 °C/0.5 h + 500 °C/8 h	1561.3	-	1.3	-
820 °C/0.5 h + 520 °C/8 h	1541.7	-	1.7	-
820 °C/0.5 h + 540 °C/8 h	1487.0	-	1.9	-
820 °C/0.5 h + 560 °C/8 h	1402.7	-	1.7	-
840 °C/0.5 h + 500 °C/8 h	1571.0	-	1.2	-
840 °C/0.5 h + 520 °C/8 h	1520.0	-	1.4	-
840 °C/0.5 h + 540 °C/8 h	1496.5	-	1.5	-
840 °C/0.5 h + 560 °C/8 h	1418.7	-	2.0	-
Ti-3Al-5Mo-5V-4Cr-2Zr (Ti-B19) [10]	1175–1360	1120–1325	7–10	17–32.5
